# The effect of coconut coir substrate on the yield and nutritional quality of sweet peppers (*Capsicum annuum*) varieties

**DOI:** 10.1038/s41598-023-29914-0

**Published:** 2023-02-15

**Authors:** Roger B. Tuckeldoe, Mdungazi K. Maluleke, P. Adriaanse

**Affiliations:** 1 Department of Agriculture, Land Reform and Rural Development, Tshwane, 0001 South Africa; 2 Department of Environmental Sciences, College of Agriculture and Environmental Sciences, University of South Africa, Tshwane, 0002 South Africa; 3 Laboratories and Horticulture Centre, College of Agriculture and Environmental Sciences, University of South Africa, Tshwane, 0002 South Africa

**Keywords:** Biotechnology, Plant sciences

## Abstract

The industry standard for estimating the quantity of horticultural produce harvested is crop yield. Producing sufficient amounts of high-quality biochemical ingredients crops can therefore aid in resolving concerns with food security and nutrition. Most producers prefer the use of organic substrate over natural soils when growing crops such as peppers in greenhouses, to ensure that there is a sufficient supply of food all year round. The objective of the study was to determine the effect of coconut coir substrate on the yield and biochemical constituents of peppers varieties grown under greenhouse environment. For two successive seasons [2021 and 2022], two sweet pepper types (Sondela and Ilanga) were cultivated on fertigated coconut coir and loamy soil (control). Fruit number, together with their dry weight and some biochemical constituents, were examined. To evaluate the impact of coconut coir substrate on the growth, yield, and biochemical constituents of different pepper cultivars grown in a greenhouse, dry plant materials and freeze-dried fruit samples were analyzed. Results showed that the coconut coir and variety (Ilanga) treatment combination produced more fruits than other treatments. Biochemical constituents such as vitamins, total phenols, total flavonoids, copper, iron and Zinc were in fruits grown under coir substrate when compared to loamy soil (control). Therefore, farmers are encouraged to grow sweet peppers varieties under coconut coir substrate for better yield, nutritional quality and profit maximisation.

## Introduction

The ever-increasing global population necessitate that food growers must look for sustainable way of producing food in order to meet the demand and supply^1^^,^^2^. The use of substrate for greenhouse crop cultivation to ensure that there is adequate food supply has been preferred by most growers as opposite to natural soils^3^. There is substantial literature on the effect of various growth media on growth, development and yield of various crops. For instance, authors such as^4,5^, is of opinion that producing food that is very nutrient-dense should be prioritized as much as improving the output of agricultural commodities, while preserving the environment, given that the majority of people adhere to a particular diet in order to improve their health. One typical practice that growers should encourage is the constant discovery and use of new organic substrate because of its benefits to the environment. One such growth media that have promising potential benefits on the growth, development and yield is coconut coir substrate^6^^,^^7^. Defined coir as the organic naturally product derived from the external husk or mesocarp of coconut fruit and has environmental benefits since its renewable. The advantage of coconut substrate has been explained by^8^ being: (i) almost 100% organic and its renewable, (ii) good drainage, (ii) high water holding capacity, (iv) accelerate root growth and development and (v) its relatively affordable. However, its effect on the growth, development, yield and quality on high valued crops agricultural crops such as peppers verities is not yet known. Research on the impact of coconut coir has not be extensively paid attention to, instead there is a lot of work on the other substrate such as compost, potting soil and sandy loam^9^. A wise grower will invest much thought and resources into selecting a suitable growth media that yield into better economical returns^10^^,^^11^. Plant roots require enough oxygen for better absorption of water and nutrients required by plants for growth, development and yield. Consequently, most growers will choose growth media that will require less irrigation frequencies and fertilizer regime^12^^,^^13^. Growing a plant in a container is different from growing in the field^14^. The optimum growth media must be able to provide plants roots with adequate moisture, air, pH and nutrients to support crop growth and yield. The objective of the study was therefore, to investigate the effect of coconut coir on the growth and yield of greenhouse grown peppers varieties.

## Method and material

### Study site and experimental set up

**Figure 1:**
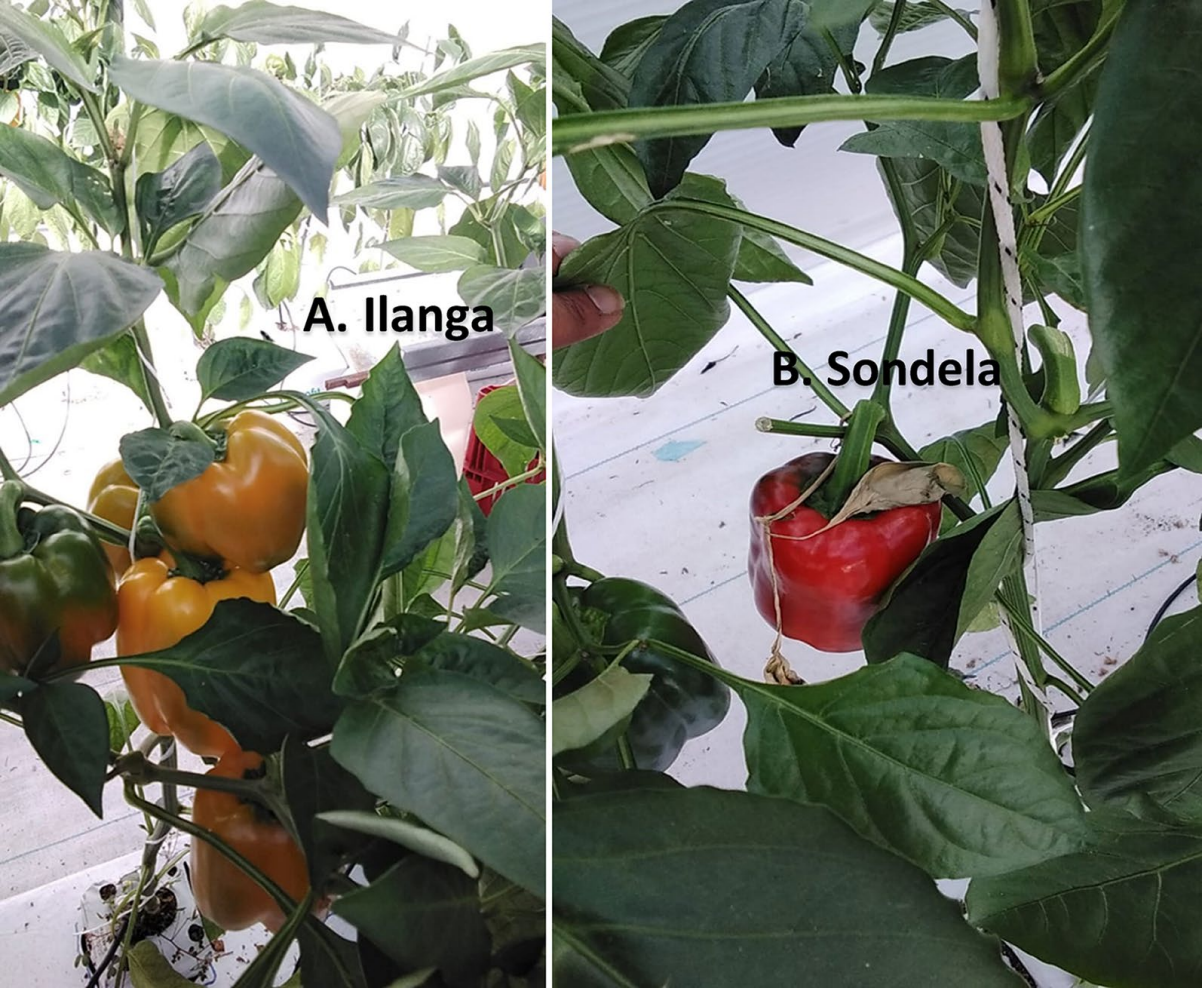
depict peppers varieties grown on coconut coir and loamy soil (control) substrate. (**A**) means variety (Ilanga); (**B**) means variety (Sondela).

In this study, sterilized growth media (coconut coir) were used. In addition, well-established healthy *Capsicum annuum* varieties (Sondela and Ilanga) seedling were sourced from a Company called (RUK ZWAAN, Krugersdorp). A factorial experiment with one factor was conducted during [2021 and 2022] seasons in a greenhouse environment at the Florida Science campus of the University of South Africa (− 26.157831 S, 27.903364 E)—sweet peppers varieties (Sondela and Ilanga, Fig. 1) were used for the experiment. Coconut coir growing bags were sourced from (Van der Knaap, South Africa). The experiment was a completely randomised design with (18) replicates. The pots were spaced 1 m apart, and an up-rope vertical trellising was used to support the plants. On each site, growing bags were either filled coconut coir media or loamy soil (control). Each block comprised 18 plants, resulting in 36 plants on the site. Each site had plants used as guard plants. Well established, uniform and healthy sweet peppers seedlings (Sondela and Ilanga) that were 30 days old, were transplanted into planting/growing bags:

$$\begin{gathered} V = Length\left( L \right) \times Width \left( W \right) \times Height \left( H \right) \hfill \\ L = 100 \,{\text{cm}} \times W = 15\,{\text{cm}} \times H = 25 \hfill \\ V = 11 250 \,{\text{cm}}^{3} \hfill \\ \end{gathered}$$

Data on plant growth parameters was collected during different stages. It is crucial to note that the experiment was conducted strictly in compliance with UNISA, the College of Agriculture and Environmental Sciences Research and Higher Degree Committee (Ethical number/Reference #: 2021/CAES HREC/136), and other local and global relevant regulations.

Prior to plant cultivation, the mineral and/or chemical composition of soil samples from coconut coir and loamy soil was analyzed (Table 1), using the method followed by^15^. The above analysis was done at the Agricultural Research Council, Institute for Soil, Climate and Water (ARC-ISWC) in Pretoria (25° 44′ 19.4″ S28° 12′ 26.4″ E).

**Table 1:** Mineral analysis for substrates used for experiment.

Chemical analysis (Micro and Macro) minerals					
	Fe	Mn	Cu	Zn	
	mg kg^−1^	mg kg^−1^	mg kg^−1^	mg kg^−1^	
Coir power	40	10	5	10	
Loamy soil	33.2	59.3	12.4	8,5	

	P	Ca	Mg	K	Na
	mg kg^−1^	mg kg^−1^	mg kg^−1^	mg kg^−1^	mg kg^−1^
Coir power	6	22	7	117	46
Loamy soil	27.8	1390	148	223	56.4

## Data collection

### Chlorophyll

Chlorophyll content was measured at different growth stages (pre-flowering, flowering and fruiting) during the experimental period. The leaf chlorophyll content (µmol/m^2^) was measured in the morning using a leaf chlorophyll meter (OPTI-SCIENCES-CCM 200 PLUS, USA). The instrument records four (4) replicate readings of the adaxial or upper leaf surface, since chlorophyll activities are more dominant on the upper leaf surface when compared to the lower surface^16^ and gives the average value.

### Stomatal conductance

Stomatal conductance (mmol m^−2^ s^−1^) was measured at different growth stages (pre-flowering, flowering and fruiting) during the experimental period. The abaxial or lower leaf surface was measured, due to the fact that stomatal opening and conductance activities are more dominant on the lower leaf surface when compared to the upper surface^17^. The porometer (Delta-T Device, AP4 Leaf Porometer, United Kingdom) was used for the measurement of stomatal conductance.

### Total biomass and above-ground plant biomass

Above-ground fresh biomass (stem, leaves and fruits) was weighed at the end of the experiment using an electronic scale (Uni-Bioc, China). The plant materials that had already been counted were weighed, placed in paper bags and in an oven for 72 h at 80 °C before re-weighing to determine dry weight. Total biomass was determined using the formula below:

1 $${\text{Total}}\,{\text{ biomass}} = {\text{ above - ground }}\,{\text{biomass }}\left( {{\text{dry}}} \right) \, + {\text{ fruit}}\,{\text{ biomass }}\left( {{\text{dry}}} \right)$$

### Fruit number and length

Number of fruits were visually counted, and fruit lengths were measured (using a 30 cm ruler) at the end of the experiment.

### Harvest index

The *C. annuum* harvest index was determined by adopting the formula used by^12^ below:

2 $${\text{HI}} = \frac{{{\text{fruit }}\,{\text{dry }}\,{\text{biomass}}\, \, \left( {{\text{dry}}} \right)}}{{{\text{total}}\,{\text{ biomass }}\,\left( {{\text{dry}}} \right)}}$$

### Water content

The formula used by^17^ was used to determine the water content for *C. annuum* crop.

3 $${\text{WC}} = {\text{ Fresh }}\,{\text{fruits }}\,{\text{weight }}\left( {\text{g}} \right) \, {-}{\text{ Dry }}\,{\text{fruits}}\,{\text{ weight}}$$

### Crude protein determination

A sample of freeze-dried fruit weighing 0.2 g was weighed, duplicated, and then analyzed using a crude protein analyser (Trumac CN-Leco, Germany). The Dumas technique is used to calculate the carbon and nitrogen percentages per 100 g. To convert nitrogen to protein, the universal protein factor 6.25 was utilized, as previously described by^18^. Ethylenediaminetetra-acetic acid was used to calibrate the Trumac CN analyser (EDTA). Glycine was utilized as a certified reference material for quality monitoring.

### Total soluble sugars determination

Peppers varieties fruit harvested from greenhouse were analysed for total soluble sugars concentration (°Brix) following the method by^19^. The fruit was cut into two portions, then juice was squeezed from a fruit portion by hand to release about 0.03 mL juice onto the aperture of the hand refractometer (HI 96,801 Refractometer, USA) and readings were taken immediately. About 18 fruits were measured per treatment/variety. The aperture was washed between different juice samples with distilled water and dried with a soft paper towel.

### Total flavonoids determination

The aluminium chloride colorimetric method reported by^20,21^ with slightly modification (triplicate), was followed to quantify pepper variety fruit samples. In a nutshell, 50 mg of fruit powder (1 mg/mL ethanol) were dissolved in 1 mL methanol, mixed with 4 mL distilled water, and then 0.3 mL of 5% NaNO2 solution; after 5 min of incubation, 0.3 mL of 10% AlCl3 solution was added, and the mixture was allowed to stand for 6 min. After adding 2 mL of 1 mol/L NaOH solution, the total volume of the mixture was brought to 10 mL using double-distilled water. The absorbance was measured at 510 nm after the mixture had been allowed to sit for 15 min. The total flavonoids content was reported in mg catechin equivalents (CE) per dry weight, with catechin used as the calibration standard.

### Total phenols determination

Method used by^22^ was adopted for the determination of total phenolic content of fruit samples, with minor modifications (triplicate). For an extraction of total phenolic content, the total phenol concentration of freeze-dried sweet peppers fruit was employed, with gallic acid as a reference (Sigma, St. Louis, MO). Folin Ciocalteu reagent (2 N, Sigma, St. Louis, MO) was used to oxidize an aliquot of the extract in a 10:1 volume/volume ratio. Samples were incubated in 96-well microplates for 20 min at room temperature, and absorbance was measured in a microplate reader at 750 nm (Synergy HT, Bio-Tek, Winooski, VT). The amount of total phenolic content was measured in mg garlic acid equivalents (GAE) per gram of dry weight (DW).

### Vitamin C and E determination

The fruit samples were freeze-dried for 72 h using a freeze drier (HARVEST-RIGHT, Barcelona). The freeze-dried fruit slices were rigorously homogenized using a sterilised food blender and mixed with dried powder before nutritional analysis. The method described by^22^ was followed with slight modifications (triplicate). Individual samples were weighed (1 g) into tube, followed by the addition of 5% metaphosphoric acid (10 ml). It was sonicated 15 min before centrifuging and filtration in the ice-cold water bath. The analysis was carried out on the model system described above, Prominence-i HLCP-PDA. A C18 Luna^®^ column (150/4.6 mm, 5 μl) held at 25 μC was used to achieve chromatographic separation. A water-based isocratic mobile phase: acetonitrile: formic acid (99:0.9:0.1) was used at a flow rate of 1 mL/min. The volume of injection was 20 μ1 and 245 nm of detection was set. Depending on the calibration curve plotted using L-ascorbic acid, sample quantification was achieved.

### Micro-nutrients determination

Freeze dried fruit samples were digested in a diffused microwave system (MLS 1200 Mega; Milestone S.r. L, Sorisole, Italy) and samples further congelated-dried following the procedure described by^23^ with minor modifications. The modifications were that samples were measured in three (3) replicates per treatment (around 15–25 mg) weighed into polytetrafluoroethylene vessels and 2 ml HNO_3_ (67%, analphur) and 1 ml H_2_0_2_ (30%, analytical grade) added in the vessels. Every solution was diluted to 15 ml in a deionized water test tube after digestion and analysed by Inductively Coupled Plasma-Mass Spectrometry (ICP-MS). An ICP-MS (Agilent 7700; Agilent Technologies, Tokyo, Japan) based on quadrupole mass analyser and octapole reaction system (ORS 3), was used to conduct the analysis. Nutrient elements such (*β*-carotene), iron **(Fe)**, copper **(Cu)**, manganese **(Mn)**, and zinc **(Zn)**, were analysed**.** The calibration solution was prepared by appropriate dilution of the single element certified reference material with 1.000 ± 0.002 g/L for each element (Analytika Ltd, Czech Republic) with deionised water (18.2 MΏ cm, Direct-Q; Millipore, France). Measurement of accuracy was verified by using certified reference material of water TM-15.2 (National Water Research Institution, Ontario, Canada).

### Data analysis for yield component

Generalised linear mixed model procedures for GenStat (version 14, VSN, UK) were used for data analysis. The model was used to assess the fixed effects of coconut coir on the growth, yield and biochemical constituents of different pepper varieties during different seasons/years on the studied variables. Significant differences for one factor (different pepper varieties (Sondela and Ilanga) were considered and reported under results section to determine the effects of all studied variables (aboveground biomass, chlorophyll content, harvest index, total biomass, stomatal conductance, fruit number, water content). Shapiro Wilk’s and Bartlett’s test were used to check the normality and homogeneity of variance. All statistical analysis was done using GenStat (version 14, VSN, UK).

### Data analysis for biochemical constituents

Data on the effect of coconut coir and loamy soil substrates on nutritional composition of peppers varieties fruit on the study variables (crude protein, total soluble sugars, total flavonoids and total phenols) were analysed using a one-way ANOVA analysis. All study variables were tested at (*P* ≤ 0.05) significance level and Duncan multiple range test was used for separation between treatment means at *P* ≤ 0.05 (95% confidence level) significant test. For all statistical analysis, Statistica v. 10, StatSoft (USA) was used.

**Table 2:** Nutrient composition in fertigation/water.

Fertigation treatment		Injection rates	
Product	Application rate	L per 1000 L	
	1000 L tank	Pre 3rd truss flower	Post 3rd truss
Tank A			
Calcium Nitrate	80 kg	5	5
Iron 6% EDDHA-chelate	1 kg	5	5
	8.10%		
Tank B			
MAP	36 kg	5	5
Tank C			
Magnesium Nitrate	80 kg	5	5
Micromix with Iron 11% DTPA-chelate	4 kg		
	8.40%		
Tank D			
Potassium sulphate	90 kg	5	5
	9.00%		
Tank E			
Nitric Acid	8L		
	11.90%		
Calculated EC (mS cm^−1^) at specified injections =		1.55	1.8

Throughout the experimental period, the computerized system (Priva, Netherlands), managed the irrigation timing and frequency as shown in Table 2.

## Results

Table 3 present the effect of coconut coir on the chlorophyll content, stem diameter and plant height of pepper varieties. Results showed that there was no significant (*P* > 0.05) difference on the chlorophyll content of peppers varieties grown under both coconut coir and loamy soil (control) during different seasons [2021 and 2022]. However, during the 2021 season, chlorophyll content ranged from 41.3 to 53.5 µmol m^−2^, while 2022 season ranged from 40.7 to 48.3 µmol m^−2^. Results showed that the treatment combination of loamy soil (control) and variety (Sondela) increased chlorophyll content from 41.3 to 53.5 mol m^−2^, but the combination of coconut coir substrate and variety (Ilanga) decreased it from 53.5 to 41.3 mol m^−2^.

**Table 3:** Effect of coconut coir on the chlorophyll content (µmol m^−2^), stem diameter (mm) and plant height (cm) of peppers varieties.

Treatment	Chlorophyll content (µmol m^−2^)		Stem diameter (mm)		Plant height (cm)	
	2021	2022	2021	2022	2021	2022
Loamy soil (control) and variety						
Ilanga	46.9 (10.1)	42.3 (7.1)	11.41 (4.8)	10.2 (0.8)	72.4 (4.9)	55.1 (11.1)
Sondela	53.5 (11.4)	42.6 (8.2)	11.81 (6.3)	9.9 (2.1)	70.9 (10.5)	54.9 (9.6)
Coir and variety						
Ilanga	41.3 (6.3)	40.7 (12.1)	10.12 (2.1)	8.96 (3.2)	64.9 (6.5)	44.3 (6.2)
Sondela	49.6 (7.1)	48.3 (8.4)	8.25 (0.5)	10.19 (3.4)	62.8 (4.1)	44.09 (7.8)
Grand mean	45.63	45.63	10.10	10.10	58.69	58.69
LSD0.05	6.04	6.04	1.04	1.04	2.21	2.21
*P* value	0.745	0.745	0.001	**0.001**	**0.001**	**0.001**

2021 means year one; 2022 means year two. Numbers in brackets represent the standard deviations of the mean. LSD_0.05_ is the least significant difference of means. *P* values in bold are lower than 0.05. LSD_0.05_ is the least significant difference of means.

For stem diameter and plant height, study results evinced that there was significant (*P* ≤ 0.05) different on the effect of substrate on peppers varieties. During [2021] season, steam diameter ranged from 8.2 to 11.8 mm, while [2022] season ranged from 8.9 to 10.2 mm. The study results delineated that treatment combination of coconut coir and variety (Sondela) reduced stem diameter from 11.8 to 8.2 mm. In comparison to other treatments, the combination of loamy soil (control) and variety (Sondela) had a stem diameter that was 11.8 mm wider.

Concerning the plant height, results showed that season one [2021] height was higher than that of season two [2022]. During season one [2021], plant height ranged from 62 to 72 cm, whereas season two plant height ranged from 44.1 to 55.1 cm. In addition, results outlined that treatment combination of coconut coir and variety (Sondela) reduced plant height from 72 to 44.1 cm, whereas treatment combination of loamy soil and variety (Ilanga) increased it from 44.1 to 72 cm.

### Fruit number

**Figure 2:**
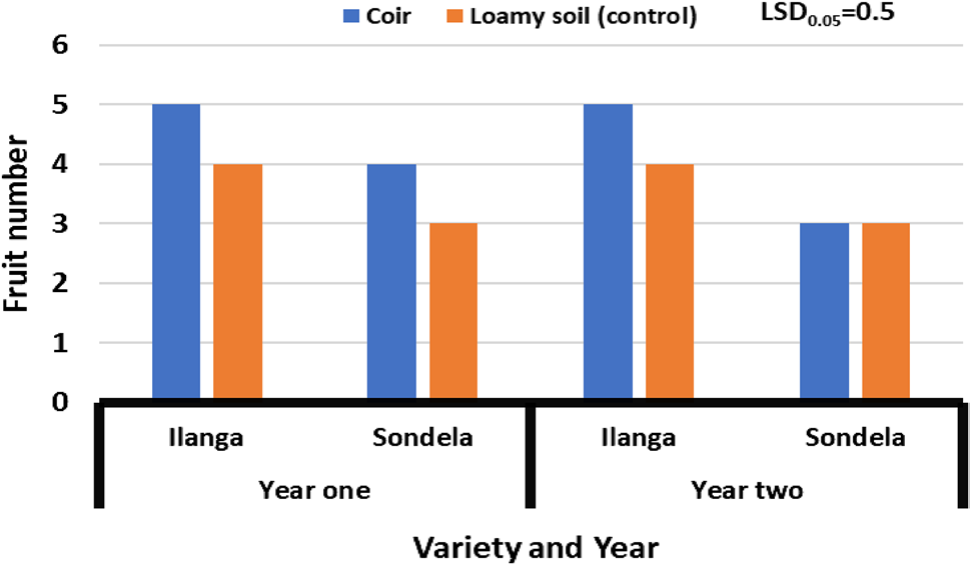
Effect of coir on the fruit number of sweet peppers varieties grown under greenhouse environment. Values are average over treatment; varieties (Ilanga and Sondela). 2021 means year one; 2022 means year two. LSD_0.05_ is the least significant difference of means.

Results in Fig. 2 revealed that there was significant (*P* ≤ 0.05) variation in fruit number on different peppers varieties grown on coir substrate. Results from season one [2021] showed that the range of fruit numbers was 3 to 5. A similar pattern was observed in season two [2022]. Treatment combination of both substrates (loamy soil-control and coconut coir) and variety (Sondela) reduced fruit number from 5 to 3, whereas treatment combination of coir substrate and variety (Ilanga) increased it from 3 to 5.It is worth to that the variety (Ilanga) demonstrate higher fruit number during both seasons [2021 and 2022].

### Total biomass

Results in Table 4 depicted that there was no substantiated (*P* > 0.05) difference on the total biomass of peppers varieties grown under different substrates (coconut coir) and loamy soil-control) during both seasons [2021 and 2022]. However, total biomass ranged from 0.10 to 0.15 kg during season [2021], while season two [2022] biomass ranged from 0.08 to 0.26 kg. The treatment combination of loamy soil (control) and variety (Sondela) reduced total biomass from 0.26 to 0.08 kg, whereas coir substrate combined with variety (Ilanga) increased total biomass from 0.08 to 0.26 kg.

**Table 4:** Effect of coconut coir on the total biomass, aboveground biomass and harvest index.

Treatment	Total biomass (kg)		AGB		Harvest index	
	2021	2022	2021	2022	2021	2022
Loamy soil (control) and variety						
Ilanga	0.14 (0.02)	0.13 (0.02)	0.08 (0.02)	0.08 (0.01)	0.42 (0.02)	0.39 (0.01)
Sondela	0.10 (0.03)	0.08 (0.01)	0.06 (0.01)	0.04 (0.01)	0.43 (0.03)	0.47 (0.02)
Coir and variety						
Ilanga	0.15 (0.01)	0.16 (0.02)	0.08 (0.01)	0.09 (0.01)	0.43 (0.01)	0.42 (0.1)
Sondela	0.13 (0.02)	0.26 (0.01)	0.07 (0.01)	0.06 (0.02)	0.42 (0.1)	0.44 (0.3)
Grand mean	0.14	0.14	0.07	0.07	0.44	0.44
LSD0.05	0.10	0.10	0.02	0.02	0.04	0.04
*P* value	0.418	0.418	0.410	0.410	0.138	0.138

2021 means year one; 2022 means year two. Numbers in brackets represent the standard deviations of the mean. *P* values in bold are lower than 0.05. LSD_0.05_ is the least significant difference of means.

### Aboveground biomass

Regarding aboveground biomass of peppers varieties grown on different substrates (coconut coir and loamy soil-control), the study results on Table 4 illustrated that there was no significant (*P* > 0.05) difference during both seasons [2021 and 2022]. However, above ground biomass ranged from 0.06 to 0.08 kg during season one [2021], whereas season two [2022] aboveground biomass ranged from 0.04 to 0.09 kg. The treatment combination of loamy soil-control and variety (Sondela) reduced aboveground biomass from 0.09 to 0.04 kg, whereas coir substrate combined with variety (Ilanga) increased it from 0.04 to 0.09 kg.

### Harvest index

Concerning the harvest index, results in Table 4 show that there was no significant (*P* > 0.05) difference on peppers grown on substrates (coir and loamy soil-control) during both seasons [2021 and 2022]. However, harvest index ranged from 0.42 to 0.45 during season one [2021], whereas season two [2022] harvest index ranged from 0.39 to 0.47. Furthermore, the study results showed that treatment combination of loamy soil-control and variety (Ilanga) reduced harvest index from 0.47 to 0.39, whereas treatment of loamy soil combined with variety (Sondela) increased it from 0.39 to 0.47.

### Water content

**Figure 3:**
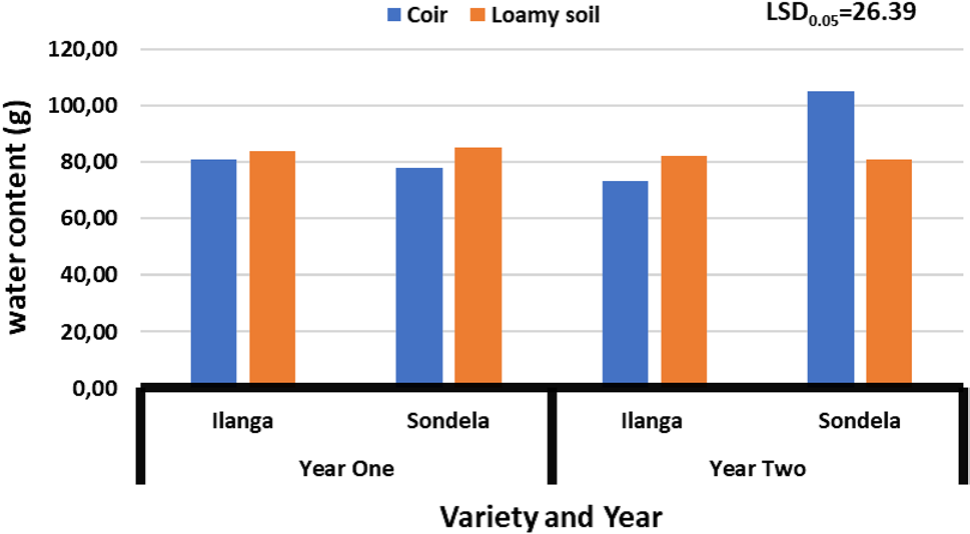
Effect of coir on the water content of sweet peppers varieties grown under greenhouse environment. Values are average over treatment; varieties (Ilanga and Sondela). 2021 means year one; 2022 means year two. LSD_0.05_ is the least significant difference of means.

Figure 3 present the effect of substrate (coconut coir and loamy soil-control) on the water content of peppers varieties during different seasons [2021 and 2022]. The study results reveal that there was no significant (*P* > 0.05) difference on the water content of peppers varieties grown on substrates (coconut coir and loamy soil-control). However, water content during season one [2021] ranged from 77.8 to 83.7 g, while season two [2022] water content ranged from 73.2 to 105 g. Moreover, results depicted that treatment combination of coconut coir and variety (Ilanga) reduced water content from 105 to 73.2 g, whereas the treatment of coconut coir combined with variety (Sondela) increased it from 73.2 to 105 kg.

Table 5 depict the effect of coir on the biochemical constituents of different sweet peppers varieties grown under greenhouse environment. Results showed that there was no significant (*P* > 0.05) difference on crude protein and total soluble sugars content on sweet peppers varieties grown under substrate coconut coir and loamy soil (control) during different seasons [2021 and 2022]. However, crude protein content during season one [2021] ranged from 6.2 to 6.3%, while season two [2022], ranged from 6.1 to 6.4%. In addition, the study results outlined that treatment combination of loamy soil (control) and variety (Sondela) reduced crude protein from 6.4 to 6.1% during season two [2022], whereas treatment combination of coconut coir substrate and variety (Ilanga) increased it from 6.1 to 6.4% during similar season.

**Table 5:** Effect of coir on the on the biochemical constituents of Sweet peppers varieties grown under greenhouse environment.

Treatment	Crude protein %		Total soluble sugars (°Brix)		Vitamin C (100 g^−1^ DW)		Vitamin E (100 g^−1^ DW)	
	2021	2022	2021	2022	2021	2022	2021	2022
Loamy soil (control) and variety								
Ilanga	6.2 (1.3)	6.3 (1.2)	5.8 (1.3)	6.6 (1.1)	343.1 (53.6)	189.6 (87)	31.7 (5.6)	36.9 (6.2)
Sondela	6.2 (1.1)	6.1 (1.1)	4.8 (1.1)	5.1 (1.1)	403.1 (59.2))	363.6 (57)	57.3 (4.4)	57.6 (14.2)
Coir and variety								
Ilanga	6.3 (1.2)	6.4 (1.1)	7.6 (1.2)	8.7 (1.1)	205.2 (16)	208.1 (27)	48.81 (12.6)	49.7 (6.9)
Sondela	6.2 (1.1)	6.3 (1.1)	6.4 (1.1)	5.2 (1.2)	501.2 (64.1)	524.4 (34)	76 (8.7)	81.1 (8.8)
Grand mean	6.23	6.23	6.26	6.26	342.3	342.3	54.88	54.88
LSD0.05	1.86	1.86	2.19	2.19	38.70	38.70	3.81	3.81
*P* value	0.32	0.32	0.41	0.41	**0.01**	**0.01**	**0.01**	**0.01**

2021 means year one; 2022 means year two. Numbers in brackets represent the standard deviations of the mean. LSD_0.05_ is the least significant difference of means. *P* values in bold are lower than 0.05. LSD_0.05_ is the least significant difference of means.

Regarding vitamin C and E content on sweet pepper varieties (Ilanga and Sondela) in substrates (coconut coir and loamy soil-control), the study results showed significant (*P* ≤ 0.05) difference. Vitamin C content during season one [2021] ranged from to 403 mg 100 g^−1^ DW, while season two [2022] vitamin C content ranged from 208 to 524 mg 100 g^−1^ DW. Furthermore, results during season one [2021],evinced that treatment combination of coconut coir substrate and variety (Ilanga) reduced vitamin C content from 524 to 208 mg 100 g^-1^ DW, whereas coconut coir substrate combined with variety (Sondela) during similar season increased it from 208 to 524 mg 100 g^−1^ DW. Concerning vitamin E content, the study results showed that it ranged from 31.1 to 76 mg 100 g^−1^ DW during season one [2021], while season two [2022] ranged from 36 to 81.1 mg 100 g^−1^ DW. Additionally, the study results showed that treatment combination of loamy soil substrate (control) and variety (Ilanga) reduced vitamin E content from 81.1 to 31.7 mg 100 g^−1^ DW during season two [2022], while coconut coir substrate combined with variety (Sondela) during season one [2021], increased it from 31.7 to 81.1 mg 100 g^−1^ DW.

Table 6 present the effect of substrates (coconut coir and loamy soil-control) on micro-nutrients (beta carotene, copper, iron and zinc) of sweet pepper varieties (Ilanga and Sondela) grown under greenhouse environment. Results showed that there was no significant (*P* > 0.05) different on iron content of sweet peppers varieties (Ilanga and Sondela) grown on substrates (coconut coir and loamy soil-control) under greenhouse environment. However, there was significant (*P* ≤ 0.05) difference on beta-carotene, copper and zinc content. Beta-carotene ranged from 1.9 to 4.1 645.5 mg 100 g^−1^ DW during season [2021], while season two [2022] content, ranged from 2.5 to 3.0 645.5 mg 100 g^−1^ DW. In addition, results showed that treatment combination of loamy soil (control) and both varieties (Ilanga and Sondela) during season one [2021] reduced beta carotene from 4.1 to 1.9 mg 100 g^−1^ DW, whereas combination of coconut coir and variety (Ilanga) during season one [2021] increased it from 1.9 to 4.1 mg 100 g^−1^ DW.

**Table 6:** Effect of coir on the on the Micro-nutrients (mg 100 g^−1^ DW) of sweet pepper varieties grown under greenhouse environment.

Treatment	Calcium		Phosphorus		Potassium		Sodium	
	2021	2022	2021	2022	2021	2022	2021	2022
Loamy soil (control) and variety								
Ilanga	492.3 (62)	439.6 (49)	373.8 (53)	370.8 (58)	261.8 (44)	255.8 (74)	373.8 (69)	370.8 (63)
Sondela	455.2 (20)	460.1 (32)	469 (92)	484 (57)	398.6 (92)	487 (81)	469 (41)	484 (98)
Coir and variety								
Ilanga	488.5 (25)	467.7 (32)	565.6 (95)	429.3 (53)	297.9 (39)	364.2 (60)	565.3 (57)	429.3 (52)
Sondela	508.3 (41)	477.6 (29)	634.9 (49)	645.5 (81)	583 (63)	650 (66)	634.9 (93)	645.5 (62)
Grand mean	473.2	473.2	496.6	496.6	412.3	412.3	496.6	496.6
LSD0.05	40.07	40.07	31.7	31.7	38.4	38.4	31.7	317.0
*P* value	0.07	0.07	0.01	**0.01**	**0.01**	**0.01**	**0.01**	**0.01**

2021 means year one; 2022 means year two. Numbers in brackets represent the standard deviations of the mean. LSD_0.05_ is the least significant difference of means. *P* values in bold are lower than 0.05. LSD_0.05_ is the least significant difference of means.

For copper content, results showed that it ranged from 11.5 to 12.2 mg 100 g^−1^ DW during season one [2021], while season two [2022] ranged from 9.2 to 10.1 mg 100 g^−1^ DW. Moreover, results showed that treatment combination of loamy soil (control) and variety (Ilanga) reduced copper content from 12.2 to 11.5 mg 100 g^−1^ DW, whereas coconut coir substrate combined with variety (Ilanga) during season one [2021] increased it from 11.5 to 12.2 mg 100 g^−1^ DW.

Regarding iron content, results showed that it ranged from 5.2 to 6.9 mg 100 g^−1^ DW during season one [2021], while season two [2022], ranged from 4.8 to 6.8 mg 100 g^−1^ DW. Additionally, results delineated that treatment combination of loamy soil (control) and variety (Sondela) reduced iron content from 6.9 to 4.8 mg 100 g^−1^ DW, whereas coconut coir combined with variety (Ilanga) increased it from 4.8 to 6.9 mg 100 g^−1^ DW. Concerning zinc content, results showed that it ranged from 5.4 to 6.9 mg 100 g^−1^ DW during season one [2021], while season two [2022], ranged from 4.9 to 5.6 mg 100 g^−1^ DW. In addition, results showed that treatment combination of loamy soil (control) and variety (Sondela) reduced zinc content from 6.9 to 4.9 mg 100 g^−1^ DW during season two [2022], while coconut coir combined with variety (Sondela) increased it from 4.9 to 6.9 mg 100 g^−1^ DW during season one [2021].

### Total flavonoids

**Figure 4:**
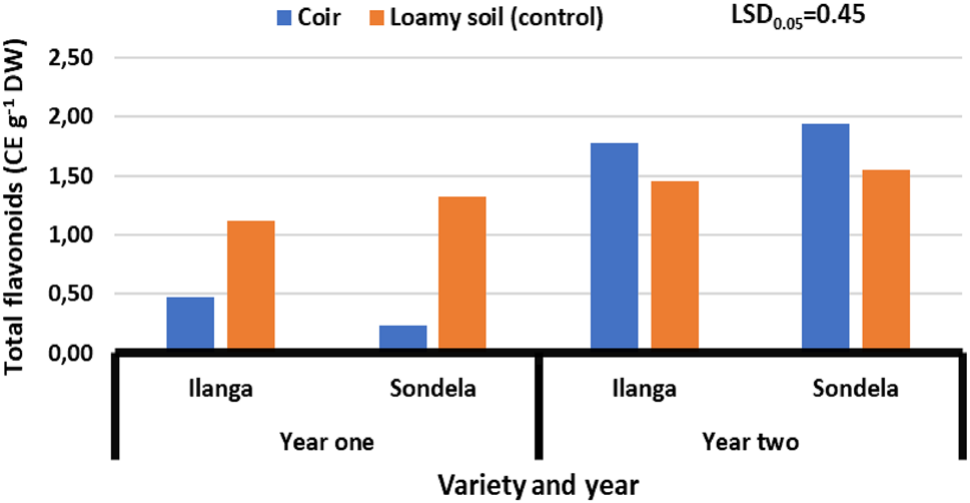
Effect of coir on the total phenolic content of sweet peppers varieties grown under greenhouse environment. Values are average over treatment; varieties (Ilanga and Sondela). 2021 means year one; 2022 means year two. LSD_0.05_ is the least significant difference of means.

Figure 4 presents the effect of substrates (coconut coir and loamy soil-control) on the total flavonoids content of sweet peppers varieties (Ilanga and Sondela) grown under greenhouse environment. Results showed that there was no significant (*P* > 0.05) difference on the total flavonoids content of sweet peppers varieties (Ilanga and Sondela) grown in substrates (coconut coir and loamy soil-control) during different seasons [2021 and 2022]. During season one [2021], total flavonoids content ranged from 0.23 to 1.33 CE g^−1^ DW, whereas season two [2022], ranged from 1.45 to 1.93 CE g^−1^ DW. In addition, results showed that treatment combination of coconut coir and variety (Sondela) reduced total flavonoids content from 1.93 to 0.23 CE g^−1^ DW during season one [2021], whereas coconut coir combined with variety (Sondela) increased it from 0.23 to 1.93 CE g^−1^ DW.

### Total phenols

**Figure 5:**
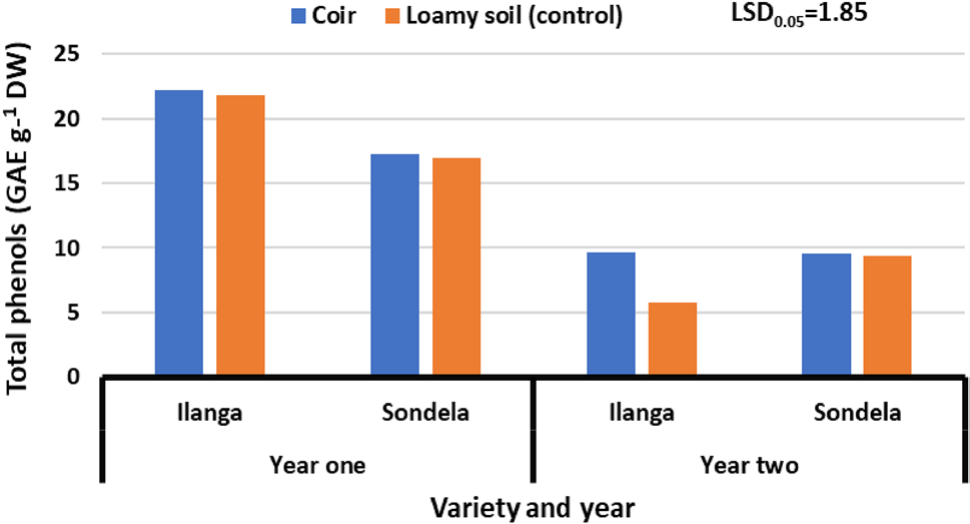
Effect of coir on the total phenolic content of Sweet peppers varieties grown under greenhouse environment. Values are average over treatment; varieties (Ilanga and Sondela). 2021 means year one; 2022 means year two. LSD_0.05_ is the least significant difference of means.

Figure 5 presents the effect of substrates (coconut coir and loamy soil-control) on the total phenols content of sweet peppers varieties (Ilanga and Sondela) grown under greenhouse environment. Results showed that there was no significant (*P* > 0.05) difference on the total phenols content of sweet peppers varieties (Ilanga and Sondela) grown in substrates (coconut coir and loamy soil-control) during different seasons [2021 and 2022]. Total phenols content ranged from 16.93 to 22.2 GAE g^−1^ DW during season one [2021], while season two [2022], ranged from 5.74 to 9.68 GAE g^−1^ DW. In addition, results evinced that treatment combination of loamy soil (control) combined with variety (Ilanga) during season two [2022], reduced total phenols content, while treatment combination of coconut coir and variety (Ilanga) during season two increased it from 5.74 to 22.2 GAE g^−1^ DW during season one [2021].

## Discussion

This study assessed the effect of coconut coir substrate on peppers varieties grown under greenhouse environment during different seasons/years. Previous research investigated the effect of nutrient management, postharvest quality, and on the yield, quality and physiological response of peppers grown on various substrate such as vermicompost and other soilless culture. According to the best of our knowledge, this is the first study to evaluate the effect of coconut coir on the growth, development and yield of different peppers varieties (Ilanga and Sondela). Therefore, the findings of this work serve as a benchmark.

### Stem diameter

Stem growth and development is associated with nutrient composition of the soil. Soil must not only provide physical support, but also adequate nutrients, water and gaseous environment for the root system to plants at all time. The study finding exhibited higher stem diameter from loamy soil-control (10.8 mm), relative to coconut coir (9.3 mm). There was 1.5 mm different between the mean values for both substrates. It has been established that macro-nutrients such as phosphorus plays a pivotal structural role in plant growth and development such as cell division and development of new tissue. Perhaps high phosphorus content in loamy soil played a crucial role in stem diameter variation. These findings concur with those of^3^, who discovered variation in plant height grown under different substrate with varying macro-nutrients content.

### Total biomass

The observations exhibit that total biomass was superior under coir substrate (0.17) relatively to the control, loamy soil (0.11). There was 0.06 kg difference between substrates. Even though the difference was not statistically noticeable, total biomass variation between these two substrates delineate that coconut coir and variety (Ilanga) was better that loamy soil (control) and variety (Sondela). Plant total biomass is linked to turgor pressure withing the guard cells. Stomatal opening occurs when solutes are actively accumulated in the guard cells. The accumulation of solutes results in a movement of water into the guard cells and a build-up of turgor pressure in excess of that in the surrounding epidermal cells. Perhaps, the fact that loamy soils have medium to poor drainage and aeration capacity could have been the possible reason for low biomass when compared to coconut coir, which has higher drainage and aeration capacity, subsequently giving it a superior ability for it to accumulate higher total biomass compared to loamy soil (control). These findings concur with those of^7^, who discovered that plants grown in coconut coir dust accumulated more biomass when compared to those grown on other substrates.

### Harvest index

In our study, findings revealed that there was slightly variation in harvest index among coconut coir (0.428) relatively to loamy soil-control (0.432). It is well known that plants ability to accumulate harvest index is linked to micro-nutrients absorption. However, that fact that loamy soil (control) micro-mineral composition was superior that of coconut coir substrates did not have any impact on harvest index on both varieties (Ilanga and Sondela). We expected loamy soil (control) harvest index to be greater than that of coconut coir substrate, but the study finding demonstrated that there was no variation among these substrates. Harvest index values obtained between substrates (coconut coir and loamy soil-control) were almost identically. Even though, their mineral composition varied significantly. Therefore, values in this study revealed that coir substrate could be a strong substrate that can be utilised for commercial growing of pepper crop in greenhouse conditions, provided that nutritional composition is well managed. These findings concur with those of^24^, who found higher yield on vermicompost compared to other substrates.

### Plant height

The study revealed that there was significant variation in plant height of both varieties (Ilanga and Sondela) between treatments. The difference in mean value between the highest loamy soil-control (63 cm) and the lowest coconut coir (54 cm) was 9 cm, with variety (Ilanga) demonstrating superior height than variety (Sondela). It is well known that calcium plays a pivotal role in plant tissues development, subsequently enable them to have better growth. Perhaps, the high calcium content in loamy soil (control) compared to that of coconut coir significantly contributed in plant height variation. Mineral composition is medium is very significant in plant growth. For example, in a study conducted by^13^, fruit yield was found to double in NPK rich substrate than that the one which had poor composition. The uptake of mineral by plants is an energy demanding process. It is well-known that calcium plays a significant role in root development, as a result, when roots are deprived from calcium, it will limit the plant ability to absorb other nutrients and negatively affect the carbohydrates ration. The study illustrated that low calcium content of coconut coir may results in shorter compared to substrates with higher calcium concentration.

### Fruit number

The grand mean evinced that fruit number was higher in coconut coir substrate relatively to loamy soil (control), with variety (Ilanga) producing more fruits than variety (Sondela). The average fruit variation was 2 among the substrates. Substrates are play a pivotal role in plant life because of their ability to provide water, nutrients, air movement within the roots for gaseous exchange. Therefore, values obtained from coconut coir could be a strong demonstration that the substrate is capable of increasing fruit number to help meet the market demands. This is important findings for farmers who specifically grow fruits for fresh market, where numbers are important in order to meet the day to day consumer demand. These discoveries are analogous to those of^25^, who found higher fruit number on well-drained substrates compared to those with poor drainage.

### Vitamin C

In plants, the primary roles of vitamin C include acting as cofactors in various metabolic pathways, facilitating the production of vital compounds for bacteria and plants, inducing pathogen resistance, directly promoting plant growth, and taking part in the conversion of compounds stored in the plant into energy^23,26^. In human health, all bodily tissues require vitamin C, also known as ascorbic acid, for their growth, development, and repair. It has a role in a variety of bodily functions, including collagen production, iron absorption, immune system function, wound healing, and cartilage, bone, and tooth maintenance^27^. The vitamin C content difference observed between the lowest (Ilanga) variety (189 mg 100 g^−1^ DW) and highest (Sondela) variety (524 mg 100 g^−1^ DW) was (335 mg 100 g^−1^ DW), representing a reasonable higher content (45 g/day) across varying age groups^28^. This means that the fruit grown variety (Sondela) grown under coir substrate has a great potential to supply human body with good amount of vitamin C, which is vital for human health. The vitamin C values of pepper variety (Sondela) under coir substrate is quite higher than those cited from^26^, likewise, that of citrus fruits reported by^29^.

### Vitamin E

Vitamin E participates in physiological processes in plants, including transpiration, which is essential for plant growth, photoprotection, photosynthesis, and yield^27^. In human health, vitamin E is a fat-soluble vitamin that comes in numerous forms, but the human body exclusively uses alpha-tocopherol. Its primary function is to act as an antioxidant, scavenging free electrons (also known as “free radicals”) that can cause cell damage^30^. Variation between content between the lowest (31.7 mg 100 g^−1^ DW) from variety (Ilanga) grown under loamy soil (control) and highest (81 mg 100 g^−1^ DW) form variety (Sondela) grown under coir substrate was (49.3 mg 100 g^−1^ DW). With these inherent values, combination of coir substrate and variety (Sondela) may be able to contribute about (30%) to vitamin E daily recommended for different age groups^27^. These finds are in harmony with those of^19^, who found variation in nutritional content of fruit harvested from varying regions on different soil types.

### Macro-nutrients

#### ꞵ-carotene

Together with chlorophylls, *ꞵ-carotene* are crucial pigments in the organs that perform photosynthetic activity^5^. In plants, *ꞵ-carotene* also serve as antioxidants, photoprotectors, colour attractants, and precursors to plant hormones. In human health *ꞵ-carotene* is transformed into vitamin A in the body (retinol). Humans require vitamin A for healthy skin and mucous membranes, as well as for good vision and eye health. Vitamin A in large levels can be hazardous, but the body only transforms as much as it needs from beta-carotene^23^. ꞵ-carotene content variation among peppers lowest (1.9 mg 100 g^−1^ DW) for variety (Sondela) grown under loamy soil substrate (control) and highest (4.1 mg 100 g^−1^ DW) for variety (Ilanga) grown under coir substrate was (2.2 mg 100 g^−1^ DW), suggesting the mineral strength of fruit grown under coir substrate. This means that fruit grown under coir substrate could play a vital role in curbing some micro-nutrients deficiencies troubling human in underdeveloped countries since its values were 38% higher than the recommended daily intake for human across all age groups as reported by^28^.

#### Copper

In plants, many enzymatic processes including the synthesis of chlorophyll and seeds production, require copper^23^. Copper deficiency can increase susceptibility to diseases, which can result in significant yield loss in most fruit crops^23^. In human health, copper helps the body make red blood cells by combining with iron. It also helps to maintain the health of blood vessels, nerves, the immune system, and the bones. Copper aids in the absorption of iron, as well. Deficiencies in this nutrient during pregnancy can cause major morphological defects in the fetus as well as long-term neurological and immunological problems in the progeny^23^.The difference between the lowest value (9.6 mg 100 g^−1^ DW) from variety (Sondela) and the highest value (12.2 mg 100 g^−1^ DW) from variety (Ilanga) under coir substrate was (2.6 mg 100 g^−1^ DW). This could mean that the substrate and variety had a direct impact on copper content. Although copper is present in soils, most of it is absorbed by plants through robust root systems, particularly in growth media such as coconut coir, which has excellent aeration.

Once absorbed, it builds up mostly in the roots, increasing the rate at which plants absorb other nutrients necessary for growth, development, and yield^28,31^. Perhaps the fact that the coconut substrate had a respectable level of copper could have been the reason why the crop produced fruits with high copper content. These findings agree with the fact that fruit nutritional content is reliant on the substrate. Therefore, growers should consider the substrate strength prior to cultivation, as this has a direct impact on the quality of produce^21^.

#### Iron

The variation between the lowest iron content (9.1 mg 100 g^−1^ DW) from variety (Sondela) grown on coir substrate and the highest (12.2 mg 100 g^−1^ DW) from variety (Ilanga) grown from similar substrate was (3.1 mg 100 g^−1^ DW). The study's findings showed that the fruit had a high chance of meeting the recommended dietary intake when grown in a greenhouse environment on balanced growth media such as coconut coir with excellent water retention capacity and good aeration. In human health, Iron is an essential component for the body's growth and development. Haemoglobin, a protein found in red blood cells that transports oxygen from the lungs to all areas of the body, and myoglobin, a protein that transports oxygen to muscles, are both made from iron. Iron is also required by the body for the production of certain hormones^29^. In human health, values obtained from this study could mean that the substrate coir, may play an important role in improving blood health related challenges such as anemia, which normally makes people get easily tired and struggle with respiration due to short of breath^1^. The study findings provide a baseline for the potential nutritional value of various horticultural crops cultivated on organic substrates such as coconut coir. Other researchers, like^8,32^, found that organic substrate like coconut has a range of positive impacts on the nutrient content of crops.

### Total flavonoids

Total flavonoids aid in cellular activity regulation and the battle against free radicals that cause oxidative stress in the body. In layman's words, they aid in the effective functioning of your body while also shielding it from everyday pollutants and stressors. Flavonoids are also effective antioxidants. The study findings revealed that the differences between the lowest total flavonoids content (0.23 CE g^-1^ DW) from the variety (Sondela) grown under coir substrate and the highest (1.9 g^-1^ DW) grown from the similar substrate was (1.67 g^-1^ DW). The change in total flavonoids may have resulted from turgor pressure in the plant cells, which subsequently permits the plant to access surrounding atmospheric elements through the epidermal cells and subsequently allow the plant to absorb atmospheric elements required by plants for cellular activities. Plant cellular activity is adversely impacted when the stomata close due to poor transpiration activities caused by imbalanced moisture from loamy soil, but it is unaffected when there is adequate water circulation within plant organs when grown under coconut coir substrate since it has a good water holding capacity and excellent aeration^23^. In human health, these values could be of immense importance in human body for diseases prevention, subsequently improving overall human health. Therefore, growing fruit on organic substrate, particularly coir, could have a positive impact in environmental sustainability and improving human health. These findings concur with those^24^, who reported that high water use efficiency and yield on greenhouse crops grown in organic substrates.

#### Total phenols

In plants, phenols typically play a role in protection against UV radiation or hostility from diseases, parasites and predators, in addition to influencing the colour of plants. Perhaps the difference in fruit number between peppers grown in coconut coir and loamy soil (control) could be explained by the fact that plants in the coconut coir group were more strongly protected from various pests and diseases due to the presence of higher phenolic compounds, which led to an increase in yield and other biochemical components^33^. In human health, phenolic compounds are important because they are potential antioxidants that prevent cell damage caused by free-radical oxidation processes.

Phenols are easily absorbed through intestinal tract walls. Humans' anti-inflammation capacity is also enhanced by phenolic acids when consumed on a regular basis^31^. The study findings revealed that the variation between the lowest (5.74 GAE g^−1^ DW) from variety (Ilanga) grown on loamy soil substrate (control) and the highest (22.2) from the same variety grown in coir substrate was (16.5 GAE g^−1^ DW). The phenolic content of fruit grown under coir substrate was reasonable high, when compared to those of loamy soil(control), indicating the coconut coir strength as a substrate that has a potential in growing healthy high nutritional crops and also could help reduce malnutrition challenges since it can improve yield^34^. These amounts of total phenols could be of vital significant in digestive process of humans by reducing constipation challenges. Similar findings were reported by^13^.

## Conclusion

The outcome of this study has shown that coconut coir combined with variety (Ilanga) and (Sondela) has a potential to be utilized as a reliable combination for commericial production under greenhouse environment. In addition, this research has provided usefull evidence to farmers, as food supply and hunger ilimination has become main focus of sustainable development goals, globally. Furthermore, this is usefull information to farmers who specifically supply fresh market, where a specific number of healthy fruits are required to meet the market demand and profit generation. In addition, most markets are geared towards organoleptic quality—in expensive markets, for example, it may be best to grow variety (Ilanga) and (Sondela) under coconut coir since its organiccaly produced. The other advantage is that the substrate can be used multiple times without harming the environment.

## Data Availability

Data generated for this study is available from the corresponding author on formal request.

## References

[1] Mashile SP, Tshisikhawe MP, Masevhe NA (2019). Indigenous fruit plants species of the Mapulana of Ehlanzeni district in Mpumalanga province, South Africa. S. Afr. J. Bot..

[2] Pingali PL (2001). Environmental consequences of agricultural commercialization in Asia. Environ. Dev. Econ..

[3] Atif MJ, Jellani G, Malik MHA, Saleem N, Ullah H, Khan MZ, Ikram S (2016). Different growth media effect the germination and growth of tomato seedlings. Sci. Technol. Dev..

[4] García-Mier L, Guevara-González RG, Mondragón-Olguín VM, Verduzco-Cuellar BDR, Torres-Pacheco I (2013). Agriculture and bioactives: achieving both crop yield and phytochemicals. Int. J. Mol. Sci..

[5] Lisiewska Z, Kmiecik W, Korus A (2008). The amino acid composition of kale (*Brassica oleracea* L. var. acephala), fresh and after culinary and technological processing. Food Chem..

[6] Adhikari P, Khanal A, Subedi R (2016). Effect of different sources of organic manure on growth and yield of sweet pepper. Adv. Plants Agric. Res..

[7] Gama PB, Wani LB, Marcelo-d’Ragga PW, Misaka BC (2015). Effect of soil media on growth of tomato seedlings (*Solanum lycopersicum* l.) under nursery (greenhouse) conditions. Int. J. Agric. Res. Rev..

[8] Olle M, Ngouajio M, Siomos A (2012). Vegetable quality and productivity as influenced by growing medium: A review. Agriculture.

[9] Unal M (2013). Effect of organic media on growth of vegetable seedlings. Pak. J. Agric. Sci..

[10] Aworh OC (2015). Promoting food security and enhancing Nigeria's small farmers’ income through value-added processing of lesser-known and under-utilized indigenous fruits and vegetables. Food Res. Int..

[11] Tomás-Callejas A, López-Velasco G, Camacho AB, Artés F, Artés-Hernández F, Suslow TV (2011). Survival and distribution of *Escherichia coli* on diverse fresh-cut baby leafy greens under preharvest through postharvest conditions. Int. J. Food Microbiol..

[12] El-Mageed TA, Semida WM (2015). Effect of deficit irrigation and growing seasons on plant water status, fruit yield and water use effeciency of squash under saline soil. Sci. Hortic..

[13] Li Y, Li J, Gao L, Tian Y (2018). Irrigation has more influence than fertilization on leaching water quality and the potential environmental risk in excessively fertilized vegetable soils. PLoS ONE.

[14] Dole JM, Wilkins HF (2005). Floriculture Principles and Species.

[15] Rahil MH, Qanadillo A (2015). Effects of different irrigation regimes on yield and water use efficiency of cucumber crop. Agric. Water Manag..

[16] Shu S, Yuan LY, Guo SR, Sun J, Yuan YH (2013). Effects of exogenous spermine on chlorophyll fluorescence, antioxidant system and ultrastructure of chloroplasts in *Cucumis sativus* L. under salt stress. Plant Physiol. Biochem..

[17] Savvides A, Fanourakis D, Van Ieperen W (2012). Co-ordination of hydraulic and stomatal conductances across light qualities in cucumber leaves. J. Exp. Bot..

[18] López A, Arazuri S, Jarén C, Mangado J, Arnal P, Ruiz I, López R (2013). Crude protein content determination of potatoes by NIRS technology. Procedia Technol..

[19] Tavarini S, Degl E, Remorini D, Massai R, Guidi L (2008). Food chemistry Antioxidant capacity, ascorbic acid, total phenols and carotenoids changes during harvest and after storage of Hayward kiwifruit. J. Food Chem..

[20] Baba SA, Malik SA (2018). Determination of total phenolic and flavonoid content, antimicrobial and antioxidant activity of a root extract of *Arisaema jacquemontii* Blume. J. Taibah Univ. Sci..

[21] Brahmi F, Mechri B, Dhibi M, Hammami M (2013). Variations in phenolic compounds and antiradical scavenging activity of *Olea europaea* leaves and fruits extracts collected in two different seasons. Ind. Crops Prod..

[22] Moyo M, Amoo SO, Aremu AO, Gruz J, Šubrtová M, Jarošová M, Doležal K (2018). Determination of mineral constituents, phytochemicals and antioxidant qualities of *Cleome gynandra*, compared to *Brassica oleracea* and *Beta vulgaris*. Front. Chem..

[23] Maluleke MK, Moja SJ, Nyathi M, Modise DM (2021). Nutrient concentration of African horned cucumber (*Cucumis metuliferus* L.) fruit under different soil types, environments, and varying irrigation water levels. Horticulturae.

[24] Jokinen K, Särkkä LE, Näkkilä J, Tahvonen R (2011). Split root fertigation enhances cucumber yield in both an open and a semi-closed greenhouse. Sci. Hortic..

[25] Van Rensburg WJ, Van Averbeke W, Slabbert R, Faber M, Van Jaarsveld P, Van Heerden I, Oelofse A (2007). African leafy vegetables in South Africa. Afr. J. Online.

[26] Parađiković N, Vinković T, Vinković Vrček I, Žuntar I, Bojić M, Medić-Šarić M (2011). Effect of natural biostimulants on yield and nutritional quality: An example of sweet yellow pepper (*Capsicum annuum* L.) plants. J. Sci. Food Agric..

[27] Achaglinkame MA, Aderibigbe RO, Hensel O, Sturm B, Korese JK (2019). Nutritional characteristics of four underutilized edible wild fruits of dietary interest in Ghana. Foods.

[28] Sharma S, Rao TR (2013). Nutritional quality characteristics of pumpkin fruit as revealed by its biochemical analysis. Int. Food Res. J..

[29] Sibiya NP, Kayitesi E, Moteetee AN (2021). Proximate analyses and amino acid composition of selected wild indigenous fruits of southern Africa. Plants.

[30] Legwaila GM, Mojeremane W, Madisa ME, Mmolotsi RM, Rampart M (2011). Potential of traditional food plants in rural household food security in Botswana. J. Hortic. For..

[31] Nwofia GE, Ojimelukwe P, Eji C (2012). Chemical composition of leaves, fruit pulp and seeds in some *Carica papaya* (L) morphotypes. Int. J. Med. Aromat. Plants.

[32] Uusiku NP, Oelofse A, Duodu KG, Bester MJ, Faber M (2010). Nutritional value of leafy vegetables of sub-Saharan Africa and their potential contribution to human health: A review. J. Food Compos. Anal..

[33] Vargas-Hernandez M, Macias-Bobadilla I, Guevara-Gonzalez RG, Romero-Gomez SDJ, Rico-Garcia E, Ocampo-Velazquez RV, Torres-Pacheco I (2017). Plant hormesis management with biostimulants of biotic origin in agriculture. Front. Plant Sci..

[34] Valverde M, Madrid R, García AL, del Amor Saavedra FM, Sánchez LR (2013). Use of almond shell and almond hull as substrates for sweet pepper cultivation. Effects on fruit yield and mineral content. Span. J. Agric. Res..

